# RNA Editing Signatures Powered by Artificial Intelligence: A New Frontier in Differentiating Schizophrenia, Bipolar, and Schizoaffective Disorders

**DOI:** 10.3390/ijms252312981

**Published:** 2024-12-03

**Authors:** Francisco J. Checa-Robles, Nicolas Salvetat, Christopher Cayzac, Mary Menhem, Mathieu Favier, Diana Vetter, Ilhème Ouna, João V. Nani, Mirian A. F. Hayashi, Elisa Brietzke, Dinah Weissmann

**Affiliations:** 1 ALCEDIAG, Parc Euromédecine, 34184 Montpellier Cedex 4, France; francisco.robles@alcediag-alcen.com (F.J.C.-R.); nicolas.salvetat@alcediag-alcen.com (N.S.); christopher.cayzac@alcediag-alcen.com (C.C.); mary.menhem@alcediag-alcen.com (M.M.); mathieu.favier@alcediag-alcen.com (M.F.); diana.vetter@alcediag-alcen.com (D.V.); 2 Sys2Diag, UMR 9005 CNRS/ALCEN, Parc Euromédecine, 34184 Montpellier Cedex 4, France; ilheme.ouna@alcediag-alcen.com; 3 Department of Pharmacology, Escola Paulista de Medicina (EPM), Universidade Federal de São Paulo (UNIFESP), São Paulo CEP 04044-20, Brazil; joao.nani@unifesp.br (J.V.N.); mhayashi@unifesp.br (M.A.F.H.); 4 National Institute for Translational Medicine (INCT-TM, CNPq/FAPESP/CAPES), Ribeirão Preto CEP 14040-900, Brazil; 5 Department of Psychiatry, School of Medicine, Queen’s University, Kingston, ON K7L 7X3, Canada; elisa.brietzke@queensu.ca

**Keywords:** schizophrenia, RNA editing, artificial intelligence

## Abstract

Mental health disorders are devastating illnesses, often misdiagnosed due to overlapping clinical symptoms. Among these conditions, bipolar disorder, schizophrenia, and schizoaffective disorder are particularly difficult to distinguish, as they share alternating positive and negative mood symptoms. Accurate and timely diagnosis of these diseases is crucial to ensure effective treatment and to tailor therapeutic management to each individual patient. In this context, it is essential to move beyond standard clinical assessment and employ innovative approaches to identify new biomarkers that can be reliably quantified. We previously identified a panel of RNA editing biomarkers capable of differentiating healthy controls from depressed patients and, among depressed patients, those with major depressive disorder and those with bipolar disorder. In this study, we integrated Adenosine-to-Inosine RNA editing blood biomarkers with clinical data through machine learning algorithms to establish specific signatures for bipolar disorder and schizophrenia spectrum disorders. This groundbreaking study paves the way for the application of RNA editing in other psychiatric disorders, such as schizophrenia and schizoaffective disorder. It represents a first proof-of-concept and provides compelling evidence for the establishment of an RNA editing signature for the diagnosis of these psychiatric conditions.

## 1. Introduction

Mental disorders are highly prevalent and often devastating diseases that negatively impact the lives of millions of people worldwide [1]. Bipolar disorder (BD) is a severe, recurrent, and often disabling mood disorder characterized by manic, hypomanic, or mixed episodes and alternating episodes of depression [2]. Within mood disorders, BD is one of the most frequent and disabling, affecting approximately 1–2% of the world’s population [3,4,5]. According to clinical data, 75% of symptomatic time for BD patients consists of depressive episodes, while manic or hypomanic episodes are less frequent. Of note, early diagnosis and treatment are associated with a more favorable prognosis [4,6]. BD is commonly underdiagnosed and often mistaken for other mental health conditions. As a result, diagnostic wandering for BD has been estimated approximately from 7 to 10 years [4,7,8,9], delaying appropriate treatment, increasing comorbidity development and suicide risk, and leading to massive costs for patients and society [10,11,12,13]. Schizophrenia (SZ) is characterized by positive symptoms such as delusions and hallucinations and negative symptoms including avolition, social withdrawal, and blunted affect [14,15]. Schizoaffective disorder (SA) is a persistent and severe illness characterized by the simultaneous manifestation of symptoms associated with SZ and affective disorders, including depression and/or (hypo)mania [2,16]. Due to symptom overlaps between BD and SZ spectrum disorders, reliable and accurate differential diagnosis represents a notable challenge for the appropriate treatment of these mental disorders [16,17,18]. Currently, the diagnosis of these pathologies is a complex and time-consuming process that relies on clinical assessment of self-reported symptoms, medical history, and clinician expertise, which is subjective and error-prone [19]. Accurate and reliable biological prediction for diagnosing fluctuating mood disorders remains an unmet clinical need, and molecular biomarkers could serve as a crucial foundation for diagnosis. Precision medicine aims to tailor medical interventions to individual characteristics identified by various “omics” approaches, including epigenomics [20,21,22]. Adenosine-to-Inosine (A-to-I) RNA editing is a post-transcriptional modification that consists of the deamination of an Adenosine (A) nucleotide into an Inosine (I) nucleotide at precise locations along the RNA molecule [23,24,25,26,27]. Depending on the location where RNA editing occurs along the RNA molecule, this mechanism can impact splicing, affect RNA stability, modulate gene expression, or directly modify protein structure and function [28,29,30,31]. This highly dynamic process enhances gene diversity and is linked to regulatory systems in psychiatry. A growing number of studies indicate that dysregulation of RNA editing is associated with neuropsychiatric disorders [28,32,33,34,35,36,37]. We have successfully identified blood RNA editing-related gene modifications that enable the differentiation with high accuracy between healthy controls and depressed patients [17], as well as between patients with BD and unipolar depression [17,38]. Recently, we further demonstrated the diagnostic value of a panel of eight RNA editing genes for BD in another cohort of Brazilian patients, using novel combinations that distinguished euthymic BD patients from depressed BD patients and healthy controls [39].

In this study, we first analyze RNA editing modifications in a panel of eight genes (*CAMK1D*, *GAB2*, *IFNAR1*, *KCNJ15*, *LYN*, *MDM2*, *PDE8A*, and *PRKCB*) across four groups of individuals (BD, SZ, SA, and healthy controls) using targeted next-generation sequencing (NGS). RNA editing biomarkers were then combined to identify the optimal signatures for distinguishing between each group. Finally, RNA editing profiles were integrated with clinical characteristics using a machine learning approach, and the diagnostic performance of the resulting algorithms was evaluated. These findings highlight the potential of artificial intelligence (AI)-based predictions using RNA-editing biomarkers to improve diagnostic precision in mental health.

## 2. Results

### 2.1. Characteristics of the Populations

This study conducted a comparative analysis involving 85 control (CTRL) subjects, 39 BD, 31 SZ, and 14 SA patients. Regarding the treatments, the following five main classes of psychiatric drugs were considered (see Table 1): antipsychotics, antidepressants, anxiolytics, antiepileptics, and hypnotics/sedatives. Additional details are available in Supplementary Material.

### 2.2. Target Editing Index (TEI)

**Figure 1:**
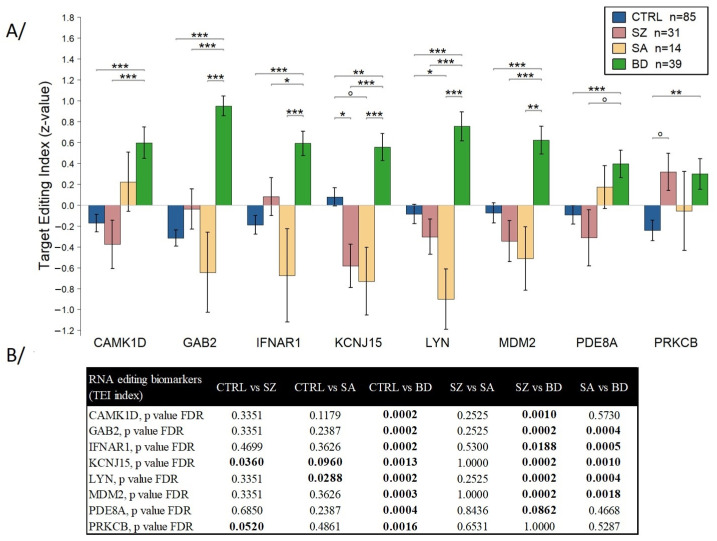
TEI analysis differentiates between the four groups (CTRL, BD, SZ, and SA). (**A**) TEI values comparing Ctrl, SZ, SA, and BD (° *p* < 0.10, * *p* < 0.05, ** *p* < 0.01, *** *p* < 0.001). (**B**) Differential TEI analysis per-group comparison, showing FDR-adjusted *p*-values (bold values are <0.1). CTRL: Control; BD: Bipolar disorder; SA: Schizoaffective disorder; SZ: Schizophrenia.

RNA editing modifications were analyzed using a panel of eight genes (*CAMK1D*, *GAB2*, *IFNAR1*, *KCNJ15*, *LYN*, *MDM2*, *PDE8A*, *PRKCB*; see details in Supplementary Material) that we previously identified and whose editing combinations were suggested as biomarkers to distinguish BD from unipolar depression [38] and subcategories of BD patients [39]. A feature selection process was implemented, incorporating the removal of highly correlated features [40], *p*-value filtering, and the Boruta algorithm [41]. This rigorous process resulted in a final list of 32 significant RNA editing biomarkers, including 3 biomarkers from *CAMK1D*, 7 biomarkers from *GAB2*, 6 biomarkers from *IFNAR1*, 3 biomarkers from *KCNJ15*, 3 biomarkers from *LYN*, 6 biomarkers from *MDM2*, 1 biomarker from *PDE8A,* and *3* biomarkers from *PRKCB* (Supplementary Figure S1). An analysis was performed to examine these 32 RNA editing biomarkers across the 4 groups, summarized as TEI (Figure 1). In comparison to the CTRL group, the SZ and SA groups showed a significant difference (*p*-value *FDR* < 0.10) in TEI on *KCNJ15* and *PRKCB* or *KCNJ15* and *LYN*, respectively. As compared to SZ and/or SA groups, the BD group revealed a significant difference (*p*-value FDR < 0.10) in TEI on *CAMK1D, GAB2*, *IFNAR1*, *KCNJ15*, *LYN*, *MDM2*, and *PDE8A*. However, no significant differences in TEI values were noticed between the SZ and SA groups.

### 2.3. RNA Editing Biomarkers Combination Analysis

The 32 significant RNA editing biomarkers were combined to assess their collective diagnostic power using two distinct approaches. First, a biomarkers-only model approach using linear combinations of RNA editing biomarkers to maximize the area under the curve (AUC) from the receiving operating characteristic (ROC) curve [42] for pairwise comparisons across all diagnostic categories. Second, an integrated model approach combined the 32 RNA editing biomarkers with covariates such as sex and psychiatric treatments, leveraging a multiclass random forest (RF) [40,43] machine learning algorithm to enhance predictive accuracy.

#### 2.3.1. Biomarkers-Only Model

**Figure 2:**
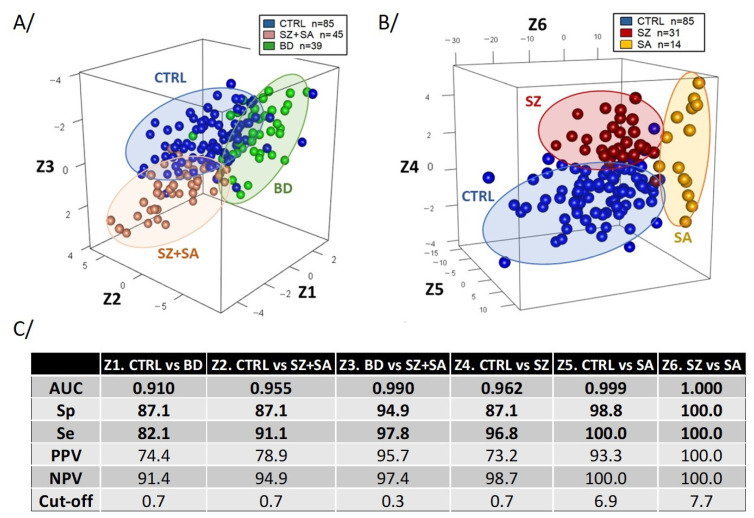
RNA editing signatures to discriminate CTRL vs. BD vs. SA vs. SZ. (**A**) Scatterplot 3D of the best combination to discriminate CTRL vs. BD vs. SZ + SA subgroups. (**B**) Scatterplot 3D of the best combination to discriminate CTRL vs. SA vs. SZ subgroups. (**C**) Table showing per-group comparison performances and decision threshold (cut-off). Z represents a combination of 32 significant RNA editing biomarkers. CTRL: Control; BD: Bipolar disorder; SA: Schizoaffective disorder; SZ: Schizophrenia; AUC: AUC ROC; Sp: Specificity; Se: Sensitivity; PPV: Positive predictive value; NPV: Negative predictive value.

Significant RNA editing biomarkers were combined to determine for each comparison the optimal linear signature, facilitating group separation. “Z” represents this combination, comprising a list of 32 significant RNA editing biomarkers. As shown in Figure 2, the combination of (Z1), (Z2), (Z3), (Z4), (Z5), and (Z6) resulted in AUC values of 0.910, 0.955, 0.990, 0.962, 0.999, and 1, with sensitivities of 82.1, 91.1, 97.8, 96.8, 100, and 100%, and specificities of 87.1, 87.1, 94.9, 87.1, 98.8, and 100%, respectively. This indicates a clear separation between CTRL and BD, CTRL and SZ + SA, BD and SZ + SA, CTRL and SZ, CTRL and SA, and SZ and SA.

#### 2.3.2. Integrated RF Model

The 32 significant biomarkers with associated covariates were combined using a multiclass RF algorithm. RF models are particularly effective on data from clinical trials because of their ability to handle complex and non-linear relationships between variables, enabling them to uncover intricate patterns and interactions within the data. The algorithm was trained on 70% of the population. Then, the test was performed on the 30% of the population who never saw the algorithm. The results obtained were plotted in a 3D scatterplot (Supplementary Figure S2A). The RF model clearly discriminates CTRL vs. BD vs. SZ vs. SA with high performance (Supplementary Figure S2A). Feature importance analysis was conducted using multiple indicators to evaluate the contribution of each feature from various perspectives. This multi-way approach ensures a more balanced assessment, mitigating the risk of relying solely on a single indicator and accounting for potential biases in feature dominance (Supplementary Table S1 and Supplementary Figures S3–S5). These analyses revealed that antipsychotics were the most influential variable in the RF model, but RNA editing biomarkers also played an important and significant role, demonstrating that they are fully integrated and utilized in the model’s predictive framework. Finally, sex was the least influential variable in the model.

Given the relatively small number of patients and the lack of an independent cohort to replicate RF results, we performed a Monte Carlo (MC) simulation to estimate prediction error (Supplementary Figure S2B). The MC method generated 500 synthetic data points from the original dataset and proved to be an effective and reliable method for data augmentation, with the synthetic data reproducing the original dataset’s structure and statistical properties (Supplementary Figures S2B and S6).

### 2.4. Results Conclusion

First, we found that RNA editing of eight previously identified genes differed significantly between healthy controls and patients with SZ and SA, as well as between BD patients and SZ/SA individuals. Conversely, RNA editing was found to be comparable between SZ and SA groups for genes considered in this study.

Then, we demonstrated that the combination of selected significant biomarkers allowed us to discriminate CTRL vs. BD vs. SZ vs. SA with high accuracy.

Finally, we showed that using machine learning algorithms to integrate these RNA editing biomarker combinations with patients’ clinical features enabled us to create an RF model with high diagnostic validity.

## 3. Discussion

Personalized medicine has become increasingly relevant to many medical fields, including psychiatry, promising earlier intervention, more efficient drug therapies, and individualized care management. BD, SZ, and SA share similar depressive symptoms and episodes of mood fluctuations [16,18], and differential diagnosis may be difficult. SA patients exhibit a clinical pattern resembling SZ, but with a longitudinal progression more akin to that of BD patients [44]. Thus, due to this diagnostic difficulty, the resulting delay is associated with inappropriate treatment and a poorer outcome [16,18,45,46]. The development of objective and reliable tools for the differential diagnosis of BD and SZ spectrum disorders is therefore essential, and several recent studies have highlighted promising research into biomarkers [47,48,49,50]. In this context, RNA editing and AI-based predictive testing represent an exciting advancement in precision medicine [17,38,39]. Machine learning, a subfield of artificial intelligence, offers the potential to significantly accelerate the diagnostic process [51]. The capacity to discriminate between these populations of patients will decrease the risk of exacerbation of the depressive state or transition to other phases (e.g., mania [52]), reduce hospitalization rates and associated costs, and diminish the comorbidities, substance abuse, and suicide risk. Furthermore, it could help maintain patients’ professional and personal lives by preventing interruptions due to ineffective care. Due to the dynamic and evolving nature of epitranscriptomic modifications, such as RNA editing, which is believed to reflect environmental cues at the molecular level, their predictive value is also expected to play a role in monitoring treatment response and emerging adverse events [53]. RNA editing analysis via NGS technology and machine learning represent an innovative approach. Previous studies have demonstrated the capacity of these techniques to efficiently identify RNA editing profiles and blood signatures with high accuracy, reproducibility, and reliability [17,38,39].

Recently, applying machine learning and combining RNA editing with clinical information from a cohort of depressed patients and healthy controls, we successfully identified specific blood signatures that demonstrated high accuracy in differentiating BD from unipolar depression (major depressive disorder, MDD) [17]. Furthermore, we validated the efficacy and reliability of this diagnostic tool using an external cohort, where an optimized algorithm achieved comparable performance in discriminating BD from MDD [38].

Additionally, we found RNA editing biomarker combinations capable of differentiating euthymic from depressed or mixed-state BD patients [39]. In this study, we extend our findings by demonstrating that specific combinations of RNA editing biomarkers from the same panel of genes can differentiate patients with BD from those with SZ and SA.

We selected a list of 32 significant RNA editing biomarkers from 8 genes used for all comparisons (Z1 to Z6, Figure 1 and Supplementary Figure S1), which led to characterizing CTRL from patients with BD, SZ, and SA (Figure 2). Using a multiclass RF algorithm, the predictive capacity of these biomarkers to differentiate CTRL, BD, SA, and SZ was tested (Supplementary Figure S2). The genes analyzed in this study are associated with specific clinical conditions, particularly psychiatric disorders and immune responses. Notably, both central and peripheral immune alterations have been observed in BD and SZ [47]. Through a series of Ca^2+^-dependent signaling cascades, CAMK1D plays a key role in neuronal development, neuronal transmission, synaptic plasticity, cognition, and neuroinflammation processes that are critical to brain function and mental health. CAMK1D promotes basal dendritic growth of hippocampal neurons, activates CREB1-dependent gene transcription factor, regulates granulocyte functions, and contributes to cytokine-induced proliferation and neutrophil activation [54,55]. Single-nucleotide polymorphisms (SNPs) in *CAMK1D* have been linked to depressive episodes and suicide attempts in patients with depression [56]. Using in vitro assays, CAMK1D was shown to phosphorylate substrates including synapsin 1 and 2, and CREB 1, which were previously associated with SZ [57]. While MDM2 is primarily recognized for its role in cancer biology [58], emerging evidence suggests it may also play a significant role in mental disorders, especially through its regulation of stress response [59], ubiquitination activity, and cell survival in the brain. MDM2 catalyzes the ubiquitination of β-arrestin, a target for antidepressants, and is involved in AMPAR surface expression during synaptic plasticity within the central nervous system [60]. Additionally, post mortem analysis revealed significant reductions in *MDM2* expression among subjects with SZ as compared to CTRL [61]. Emerging research suggests that IFNAR1 dysregulation and the resulting impact on type I IFN signaling can influence the pathophysiology of several mental disorders via neuroinflammation [62]. RNA editing shows a strong association with interferon response, and polymorphisms in the promoter region of *IFNAR1* have been linked to increased depression risk [63,64,65]. Interferon-based treatments can lead to adverse effects, including neurological and neuropsychiatric disorders, as well as autoimmune disease [62]. Emerging evidence suggests that LYN dysregulation may contribute to the pathophysiology of certain mental disorders, primarily through its involvement in immune modulation and neural signaling pathways [66]. LYN has been found to enhance glutamatergic synaptic transmission and activate the mitogen-activated protein kinase (MAPK) pathway, which increases Brain-Derived Neurotrophic Factor (BDNF) expression [67]. LYN also modulates N-methyl-D-aspartate receptor (NMDAR) function, a pathway critical to SZ, as NMDAR hypofunction is implicated as a core component of the disorder [68]. PRKCB, a member of the protein kinase C (PKC) family, is particularly important in the brain through its influence on neurotransmitter release, synaptic plasticity, and neural signaling. Further findings highlight that *PRKCB* expression is downregulated in the peripheral blood mononuclear cells of depressed patients, with specific SNPs in *PRKCB* showing association with major depression [69]. Located at 16p12.2-p12.1, *PRKCB* encodes a protein essential to synaptic plasticity [70], and impaired synaptic plasticity is increasingly recognized as a pathogenic factor in SZ [71]. In addition, abnormalities in PKC have been implicated in BD, with decreased PKC activity observed in the prefrontal and temporal cortex of BD patients [72]. Indeed, endoxifen, a PKC inhibitor, significantly improved type I BD symptoms [73]. PDE8A is a cAMP-specific phosphodiesterase that plays a vital role in cellular signaling by hydrolyzing cAMP, a second messenger regulating various physiological processes such as neuronal function and inflammation [74,75,76]. In patients with MDD, expression of *PDE8A* in the temporal cortex is reduced by half, and PDEs are known to play key roles in signal transduction, inflammatory cell activation, memory, and cognition [77]. Memory and cognitive impairments are common in SZ, further linking *PDE8A* to these psychiatric conditions [78]. GAB2 is widely expressed in the central nervous system and is a pivotal component in immune responses, engaging in multiple signaling pathways [79,80]. GAB2 overexpression makes neurons vulnerable, increasing tau phosphorylation and leading to the Alzheimer’s disease phenotype [81]. Genetic analyses also identify the DISC1 locus as significant. This gene was originally discovered in a Scottish pedigree in which chromosomal translocation (1;11) (q42.1; q14.3) was linked to multiple psychiatric illnesses, including SZ, BD, and MDD. GRB2, through its docking on GAB2, interacts with DISC1 in the brain, competing with NDEL1 (nuclear distribution element like-1) and playing a critical role in pathways involving ErbB receptors related to SZ [82]. Lastly, KCNJ15, a member of the inwardly rectifying potassium channel family, plays a role in maintaining potassium ion homeostasis. Potassium channels are involved in the regulation of neuronal excitability and neurotransmission, processes often disrupted in neurological diseases including epilepsy [83] and Parkinson’s disease [84]. KCNJ15 has been shown to influence immune activity [85], with specific variants linked to an increased risk of Alzheimer’s disease in the Chinese population, possibly acting through immune modulation [85]. Thus, all the identified genes exhibit a relationship, either direct or indirect, with neuronal and/or inflammatory mechanisms, particularly BD and SZ spectrum disorders.

Medications are included as covariates within the RF model. By incorporating in the model RNA editing biomarkers, psychiatric treatment classes (based on ATC classification), and sex as features, the model captures complex relationships among these factors and their combined influence on diagnostic categories. RF models are especially valuable for medication adjustments as they effectively manage the complex non-linear interactions often encountered in clinical trials. Additionally, the model’s ability to assess feature importance adds transparency, allowing clinicians to understand which variables most significantly influence the predictions. Psychiatric treatment ATC classes frequently appear as root variables in the decision trees, although RNA-editing biomarkers also hold prominent positions. Moreover, the results indicate that the model’s predictions are not sensitive to biological sex. Analysis of the model’s explainability suggests that the threshold values of RNA-editing biomarkers within the decision trees closely align with the influence of medications, indicating a meaningful interplay between treatment effects and biomarker responses in the model’s classification process. Furthermore, all these results demonstrate the importance of RNA editing biomarkers in the RF model and their adjustment with medications. The performances and spatial classification of the four classes obtained from simulated data using the MC method enhance the reliability of this model as a first step to large cohort validation. The MC simulation approach proves to be an effective method for data augmentation, addressing the challenges of limited data availability.

Some limitations should be considered in this study. First, patients in the BD group were in different mood states (hypomanic/manic, depressive, mixed, and euthymic bipolar). Second, sex proportion was significantly different among the groups. Third, the small sample size (particularly for the SA group) may induce a potential bias in this study, and without replication, it is important to take the results of this study with caution.

This initial proof-of-concept analysis presents compelling evidence for the establishment of an RNA editing signature for diagnosis and potentially for prognosis and/or treatment prediction. Further validation using a larger cohort and replication of these results will be required.

## 4. Materials and Methods

This study involved the following complementary methodological approaches:

Recruitment of healthy control individuals (CTRL, *n* = 85) and patients with bipolar disorder (BD, *n* = 39), schizophrenia (SZ, *n* = 31), and schizoaffective disorder (SA, *n* = 14), using 2 independent cohorts.

RNA extraction from blood samples of recruited individuals and targeted sequencing of a previously defined panel of RNA editing genes.

Bioinformatics analysis for ensuring the quality of sequencing data, filter data based on length and quality score, perform the alignment with reference human genome, and identify editing events (site, isoform, and/or pattern, referred to as biomarkers; see Supplementary Material).

Biostatistics for calculating a Target Editing Index (TEI; see below and Supplementary Material for more details) are obtained in the following way: finding significant differences in editing between groups of individuals (CTRL vs. BD vs. SZ vs. SA) and combining identified biomarkers using machine learning approaches (Random Forest algorithm), which consider patient clinical characteristics.

Additional information and references supporting the methods used in this study, particularly for bioinformatics and biostatistical analysis, are provided in Supplementary Material.

### 4.1. Subjects and Clinical Assessment

The patient samples included in this study originated from two distinct cohorts. The Helsinki Declaration’s guiding principles were closely followed throughout this study. All participants, aged between 18 and 65 years, signed a written informed consent before entering this study. The cohort of BD patients was approved by the Research Ethics Committee of UNIFESP [CEP No. 1427/16]. Samples from patients with SZ and SA were obtained from PrecisionMed Inc. These samples were collected with informed consent under Western Institutional Review Boards (WIRB^®^) protocol #20041439 and protocol no 1022, reviewed and approved by their Institutional Review Board. The Structured Clinical Interview for DSM-IV Axis I disorders (SCID-1) was conducted with eligible participants. The Hamilton Depression Rating Scale-17 items (HDRS) and the Young Mania Rating Scale (YMRS) were used to assess the severity of depressive and manic symptoms, respectively. Patients in this study were diagnosed with SZ according to M.I.N.I. (Mini International Neuropsychiatric Interview) and SCI-PANSS (Structured Clinical Interview for the Positive and Negative Syndrome Scale). A form document completed by the subjects’ psychiatrist confirmed their diagnosis according to DSM-IV criteria. Volunteers in the healthy control group included individuals without a history of psychotropic medication use, no lifetime or current mental disorders, and no family history of a major psychiatric disorder in first-degree relatives. More details about demographic and clinical characteristics are shown in Table 1.

### 4.2. RNA Extraction and Qualification

Samples were retrieved in PAXgene™ blood RNA tubes and extracted using the MagNA Pure 96 instrument (Roche, Bâle, Suisse). Total RNA concentrations and quality were determined with Qubit Fluorometer (Life technologies, Carlsbad, CA, USA) and LabChip GX (Perkin-Elmer, Waltham, MA, USA) instruments, respectively.

### 4.3. Targeted Next Generation Sequencing

A panel of blood RNA editing genes (*PRKCB*, *PDE8A*, *CAMK1D*, *GAB2*, *IFNAR1*, *KCNJ15*, *LYN*, and *MDM2*) was designed [39], and validated primers were used to amplify each gene on a Peqstar 96 × thermocycler (VWR, Radnor, PA, USA). PCR products underwent purification with magnetic beads, quantification, and indexing. The resulting library was pooled, purified, denatured, spiked with PhiX Control V3 (Illumina, San Diego, CA, USA), and loaded onto a sequencing cartridge. Finally, the library was sequenced using Illumina NextSeq 500 (Illumina, San Diego, CA, USA).

### 4.4. Bioinformatics Analysis

FastQC software (version 0.11.9) was used to verify the quality of the sequencing data. A minimal sequencing depth of 10,000 reads for each sample was considered for further analysis. A pre-treatment step was performed, assessing both length and quality scores. Bowtie2 was used to align the processed reads to the reference human genome sequence, GRCh38 [86]. For SVN calling, SAMtools mpileup was applied [87,88]. By counting the different nucleotides in each genomic region, proprietary scripts were used to identify the edited positions in the alignment. RNA editing values for all potential biomarkers (including sites, isoforms, and motifs) were evaluated against the background (for more information see Supplemental Methods in Supplementary Material).

### 4.5. Biostatistical Analysis

All statistics and figures were computed with the “R/Bioconductor” software (R version 4.4.1, Bioconductor Manager version 3.19) [89,90]. A differential analysis was carried out using the most appropriate test between the Mann–Whitney rank-sum test, Student’s *t*-test, or Welch’s *t*-test according to normality and sample variance distribution in each cohort. The false discovery rate (FDR) was managed through the use of the Benjamini and Hochberg (BH) procedure [91], and an adjusted *p*-value below 0.1 was considered statistically significant. Due to numerous significant biomarkers (*p*-value ≤ 0.05), a feature selection process was implemented by selecting the biomarkers less correlated (R = 0.6) and showing more importance related to the class, and using the functions find correlation from the R package “caret” version 6.0-94, the Boruta algorithm (a robust feature selection method based on the random forest classifier) from the R package “Boruta” version 8.0.0, and the function mutinformation (a feature selection method based on an entropy estimator) from the R package “infotheo” version 1.2.0.1 [40,41,92]. This rigorous feature selection process resulted in a final list of 32 significant RNA editing biomarkers. A “Target Editing Index” (TEI), resuming gene-specific editing values, was calculated by a linear combination of significant RNA editing variants maximizing the AUC of ROC (Figure 1) [42]. The TEI was transformed using the Boxcox transform, aiming to guarantee the normal distribution of data [93]. After assessing the target validity with the TEI analysis, we explored a signature of 32 combined RNA editing biomarkers to classify all classes. The 32 biomarkers were normalized by the corresponding target global editing and then combined using the mROC multivariate method for each comparison (Figure 2). The equation for the respective combination is provided and can be used as a new virtual marker Z, as follows: Z = a × biomarker 1 + b × biomarker 2 + c × biomarker 3, where a, b, and c are calculated coefficients and biomarkers 1, 2, and 3 are the levels of biomarker.

Finally, the same 32 biomarkers were combined using multiclass Random Forest (RF) [43], considering patient treatment and sex (see Table 1 and Supplementary Material). RF requires the use of a training set to construct the model (70% of the population; *n* = 120 samples) and a test set (30% of the population; *n* = 49 samples). This sharing has been randomized and respects the initial proportion of the various statutes in each set. The RF method combines Breiman’s “bagging” idea and the random selection of features in order to construct a collection of decision trees with controlled variance. To generalize the model, we performed 1000 learning RF trees (*n* = 120). The final classifier was generated by summing up the votes (probabilities) for each applied sample from each tree and was normalized by the number of trees. The end resulting probabilities reflect the majority vote of the 1000 RF trees. We used a grid learning approach for each individual tree, where we stated certain maximum parameter sets (ntree  =  1000, nodesize  =  1 and mtry  =  (1, 20)). RF results are shown on the test dataset (*n* = 49), which has never seen the algorithm. The implementation was performed using the R RandomForest package (version 4.6-14) and the R caret package (version 6.0-84). Variable importance analysis was assessed using the randomForestExplainer packages in R [94]. Monte Carlo (MC) simulation was performed to generate synthetic data [95]. For more information about RF model interpretability and MC simulation, see Supplemental Methods in Supplementary Material.

## 5. Conclusions

RNA editing coupled with AI paves the way for improved classification of patients with mood fluctuations into specific disease subcategories. This approach aligns with the principles of precision psychiatry, aiming to deepen our understanding of the pathophysiological mechanisms of depression and improve therapeutic care. In addition, RNA editing and machine learning represent valuable tools in attempting to think beyond diagnostic symptoms, better understand heterogeneity in psychiatric disorders, and consider the underlying features of psychopathology from a neuroscience-based approach.

## Figures and Tables

**Table 1:** Demographic and clinical characteristics of the study population. Data are presented as mean ± SEM. *p*-values for age were obtained using Student’s *t*-test, while *p*-values for sex and psychotropic treatments were calculated using the chi-square test (bold values are ≤0.05). Controls: Healthy volunteers; SZ: Schizophrenia patients; SA: Schizoaffective patients; BD: Bipolar disorder patients.

	Total Sample	Controls	SZ	SA	BD
**Number, n**	169	85	31	14	39
**Age**					
Age (min–max)	18–64	18–63	22–62	35–63	18–64
Age (mean ± SD)	42.2 ± 10.5	40.3 ± 11.2	42.8 ± 9.8	45.6 ± 6.7	44.7 ± 9.7
*p* value (vs. Ctrl)			0.24	**0.02**	**0.03**
*p* value (vs. SZ)				0.27	0.42
*p* value (vs. SA)					0.71
**Sex**					
Male (n (%))	91 (53.8)	47 (55.3)	23 (74.2)	10 (71.4)	11 (28.2)
Female (n (%))	78 (46.1)	38 (44.7)	8 (25.8)	4 (28.6)	28 (71.8)
*p* value (vs. Ctrl)			**<0.0001**	**0.001**	**<0.0001**
*p* value (vs. SZ)				0.52	**<0.0001**
*p* value (vs. SA)					**<0.0001**
**Psychotropic Treatments**					
Anxiolytics (n (%))	7(4.1)	0	5 (16.1)	2 (14.3)	0 (0.0)
Hypnotics/Sedatives (n (%))	1 (0.59)	0	1 (3.2)	0 (0.0)	0 (0.0)
Antidepressants (n (%))	31 (18.3)	0	18 (58.0)	8 (57.1)	5 (12.8)
Antipsychotics (n (%))	71(42.0)	0	29 (93.6)	14 (100.0)	28 (71.8)
Antiepileptics (n (%))	43 (25.4)	0	12 (38.7)	5 (35.7)	26 (66.7)
*p* value (vs. SZ)				0.39	**<0.0001**
*p* value (vs. SA)					**<0.0001**

## Data Availability

Readers will be able to access the data, associated protocols, code, and materials in the paper by asking to Dinah Weissmann (dweissmann@alcediag-alcen.com).

## Supplementary Materials

The following supporting information can be downloaded at: https://www.mdpi.com/article/10.3390/ijms252312981/s1. References [96,97,98,99] are cited in the supplementary materials.
