# Supplementary material for: RNA Editing Signatures Powered by Artificial Intelligence: A New Frontier in Differentiating Schizophrenia, Bipolar, and Schizoaffective Disorders
Source: Int J Mol Sci. 2024 Dec 3;25(23):12981. doi: 10.3390/ijms252312981 (PMC11641080; doi:10.3390/ijms252312981)
Supplement: Supplementary file 1 [file ijms-25-12981-s001.zip › ijms-3299986-supplementary.pdf]

Supplementary Material for

RNA Editing Signatures Powered by Artificial Intelligence: A New Frontier in Differentiating Schizophrenia, Bipolar and Schizoaffective Disorders

Francisco J. Checa-Robles, Nicolas Salvetat, Christopher Cayzac, Mary Menhem, Mathieu Favier, Diana Vetter, Ilhème Ouna, João V Nani, Mirian A.F. Hayashi, Elisa Brietzke, Dinah Weissmann

This PDF file includes:

Supplemental Methods

Supplementary Table S1

Supplementary Figures S1-S6

Supplementary Information references

Abbreviations of HGNC (HUGO Gene Nomenclature Committee) gene names:

ADAR: Adenosine deaminases acting on RNA

GAB2: GRB2 Associated Binding Protein 2

IFNAR1: Interferon Alpha and Beta Receptor Subunit 1

LYN: LYN Proto-Oncogene, Src Family Tyrosine Kinase

MDM2: MDM2 proto-oncogene

PRKCB: Protein Kinase C Beta

PDE8A: Phosphodiesterase 8A

CAMK1D: Calcium/Calmodulin Dependent Protein Kinase 1D

KCNJ15: Potassium inwardly rectifying channel subfamily J member 15

Supplemental Methods

Characteristics of the populations

In this study, 169 participants are included, comprising 85 healthy volunteers, 31 patients diagnosed with schizophrenia, 14 with schizoaffective disorder, and 39 with bipolar disorder.
The t-test was used to compare the age across different groups, while the chi-squared test was employed to compare sex and treatments among the various groups. No significant difference ($p > 0.05$) was found in the age among the different groups, except between the SA and BD groups compared to the controls ($p < 0.05$). There was a significant difference ($p < 0.05$) in sex among all groups except between the schizoaffective and schizophrenic groups. Lastly, a significant difference ($p < 0.001$) in psychiatric treatment was observed between the BD group and the SA and SZ groups (Table 1), while no significant difference ($p > 0.05$) was observed between the SA and SZ groups. To prevent a putative bias in further analyses due to differences in medications, the algorithm results were adjusted for sex and psychiatric treatments.

Bioinformatics analysis of targeted sequencing data

The sequencing data obtained from the Illumina NextSeq 500 underwent quality assessment using the FastQC software (version 0.11.7, https://github.com/s-andrews/FastQC/). A minimum sequencing depth of 10,000 reads for each sample and each target was established for further analysis. Initial processing involved removing adapter sequences and filtering based on length and quality score, with short reads (< 100 nts) and reads with an average QC < 8 being eliminated. Flexible read trimming and filtering tools for Illumina NGS data, including fastx_toolkit v0.0.14 and prinseq v0.20.4, were employed to enhance sequence alignment quality. After pre-processing steps, each cleaned fastq file underwent additional quality control before proceeding to further analysis. The processed reads were aligned using bowtie2 (version 2.2.9) [1] with end-to-end sensitive mode. The alignment was done to the reference human genome sequence GRCh38. Non-unique alignments, unaligned reads, or reads containing insertion/deletion (INDEL) were removed from downstream analysis using SAMtools software (version 1.7) [2]. SAMtools mpileup was used for SVN calling. Identification of edited positions in the alignment was performed using in-house scripts to determine the number of different nucleotides at each genomic location. For each position, the script calculated the percentage of reads containing a 'G' nucleotide [Number of 'G' reads/ (Number of 'G' reads + Number of 'A' reads)*100]. Genomic locations with a reference 'A' and a percentage of 'G' reads > 0.1 were automatically identified by the script as 'A-to-I edition site'. The final step involves calculating the percentage of all possible isoforms of each transcript. By definition, the relative proportion of RNA editing at a given editing 'site' represents the sum of editing modifications measured at this unique genomic coordinate. Conversely,

an edited mRNA isoform is a unique molecule that may or may not contain multiple editing modifications on the same transcript. For example for a given transcript, the edited mRNA isoform BC contains an A-to-I modification on both site B and site C within the same transcript. An editing pattern is one combination of editing events occurring at sites of interest in a transcript. By definition, given a list of sites of interest, a transcript has as many editing patterns as there are possible combinations. For example for a given transcript, with sites of interest ABC, the analyzed patterns are [A, B, C, AB, BC, AC, and ABC]. We take into consideration the term "biomarker" to refer to an RNA editing site, isoform and or pattern with a significant diagnostic value in at least one studied comparison.

Random Forest Model Interpretability and Importance of Variables

To comprehensively evaluate the importance of the features considered by the model, various variable importance metrics of the AI model were explored using the randomForestExplainer package [12]. This importance metrics list included: total number of trees, total number of nodes, mean minimal depth (based on the structure of the forest, where features near the root were considered more important than others), node impurity (Gini index decrease based on changes in node purity after splits on the variable), the number of times a variable was selected as a root and P-values (based on the one-sided binomial test to assess the significance of feature importance). A deeper understanding analysis of how RNA editing biomarkers interact with medication was included, using the same R package, and variables importance metrics and statistic tests values of the RF model were compiled on a table (see Supplementary Table S1).

Monte Carlo Simulation

To implement the Monte Carlo method simulation in R [13], the original dataset of 169 samples and 36 features has been used to obtain n = 500 synthetic samples, replicating the statistical properties and class distributions of the original dataset by repeated random sampling of feature values following specific probability distributions. For continuous features (biomarkers), a multivariate normal distribution was assumed (function `mvrnorm` du package MASS, version 7.3-60.2 [14]) combined with correlation method to ensure capturing inter-feature relationship. Binary features (sex and treatment regimen) followed a Bernoulli distribution. Post-processing was applied to adjust the means of continuous features for better accuracy. Validation of the simulated data involved comparing the class distribution of each dataset, as well as the comparison of statistics distribution metrics (means, standard deviations, skewness, kurtosis) of each feature.

variable	No of trees	No of nodes	Mean min depth	Gini decrease	Times a root	P_value	Sign
CAMK1D_bmk1	529	712	3,3459	2,147	30	**<0,0001**	***
CAMK1D_bmk2	388	498	3,4433	1,336	10	0,9999	ns
CAMK1D_bmk3	437	570	3,3501	1,619	9	0,7003	ns
GAB2_bmk1	529	740	3,0473	2,542	21	**<0,0001**	***
GAB2_bmk2	458	600	3,4476	1,742	17	0,2315	ns
GAB2_bmk3	423	559	3,3735	1,511	16	0,8395	ns
GAB2_bmk4	446	575	3,3879	1,557	4	0,6232	ns
GAB2_bmk5	441	578	3,4172	1,678	12	0,5743	ns
GAB2_bmk6	411	525	3,6131	1,360	4	0,9930	ns
GAB2_bmk7	413	521	3,4722	1,351	7	0,9957	ns
IFNAR1_bmk1	448	611	3,3147	1,831	19	0,1169	ns
IFNAR1_bmk2	387	502	3,4858	1,359	14	0,9997	ns
IFNAR1_bmk3	460	580	3,0739	1,916	41	0,5411	ns
IFNAR1_bmk4	436	551	3,2683	1,593	19	0,9089	ns
IFNAR1_bmk5	441	566	3,3900	1,571	5	0,7564	ns
IFNAR1_bmk6	389	486	3,4859	1,293	4	1,0000	ns
KCNJ15_bmk1	427	571	3,4333	1,499	8	0,6855	ns
KCNJ15_bmk2	480	623	3,0583	2,191	50	**0,0459**	*
KCNJ15_bmk3	530	695	2,9547	2,464	51	**<0,0001**	***
LYN_bmk1	438	563	3,4543	1,591	16	0,7945	ns
LYN_bmk2	404	522	3,2896	1,626	30	0,9951	ns
LYN_bmk3	576	791	2,9253	2,820	35	**<0,0001**	***
MDM2_bmk1	457	598	3,3501	1,794	12	0,2576	ns
MDM2_bmk2	345	418	3,9246	1,002	1	1,0000	ns
MDM2_bmk3	486	641	3,4856	2,018	17	**0,0077**	**
MDM2_bmk4	493	653	3,5132	1,942	11	**0,0018**	**
MDM2_bmk5	457	583	3,0941	1,928	44	0,4908	ns
MDM2_bmk6	402	503	3,6667	1,311	7	0,9997	ns
PDE8A_bmk1	483	610	3,1718	2,026	33	0,1252	ns
PRKCB_bmk1	493	658	3,2475	1,969	14	**0,0009**	**
PRKCB_bmk2	486	642	3,3642	1,998	16	**0,0069**	**
PRKCB_bmk3	394	517	3,6523	1,277	2	0,9975	ns
Sex	149	158	3,3087	0,509	1	1,0000	ns
ANTIDEPRESSANTS	426	459	1,8779	3,389	114	1,0000	ns
ANTIEPILEPTICS	574	627	2,0976	5,886	133	**0,0321**	*
ANTIPSYCHOTICS	792	950	2,1629	12,375	173	**<0,0001**	***

Supplementary Table S1. Variables importance metrics and statistic tests of the RF model. No of trees: total number of trees; No of nodes: total number of nodes; Mean min depth: mean minimal depth based on the structure of the model; Gini decrease: Gini index of node impurity by splits on variable; Times a root: number of times a variable was selected as a root; p_value: *p*-value based on the one-sided binomial test to assess the significance of feature importance calculated by RandomForestExplainer R package; Sign: Significance; *** p < 0,0001, ** p < 0,01, * p < 0,05; ns: non-significant.

Supplemental Figures

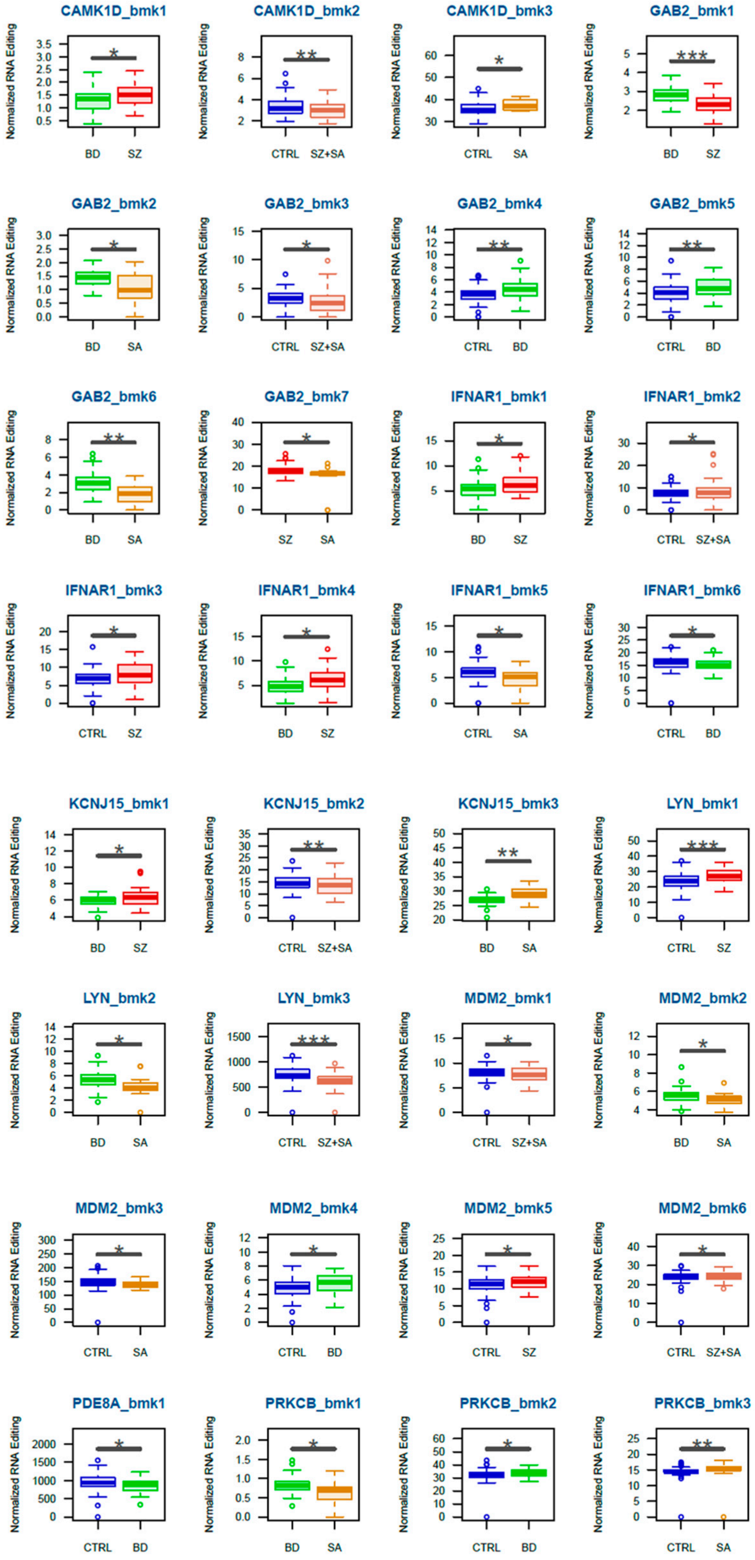

Supplementary Figure S1. **Boxplot of the 32 significant RNA editing biomarkers.** Normalized RNA Editing values for each of the 32 significant biomarkers included in the classification model. All the biomarkers were significant for at least one comparison. The figure represents an example of a comparison for each of the 32 biomarkers. CTRL: Control; BD: Bipolar Disorder; SA: Schizoaffective disorder; SZ: Schizophrenia; SZ+SA: SZ: Schizophrenia + Schizoaffective disorder.

A/

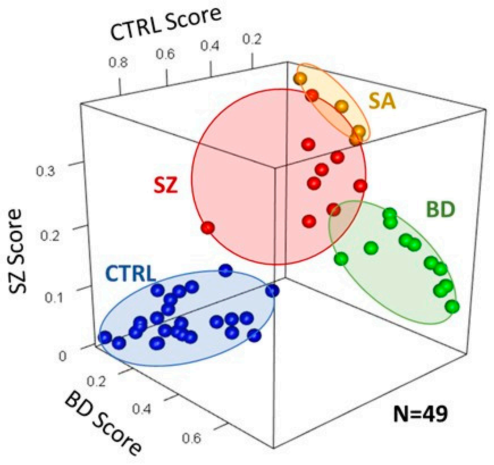

	Precision	Recall	F-1 Score
CTRL	0.93	1	0.94
SZ	1	0.89	0.98
SA	1	0.75	0.94
BD	1	1	1

Macro Average	0.98	0.91	0.97
Weighted Average	0.96	0.96	0.96

B/

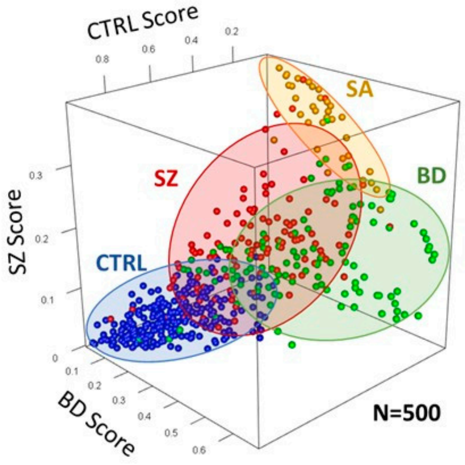

	Precision	Recall	F-1 Score
CTRL	1	0.54	1
SZ	0.8	0.94	1
SA	0.94	1	1
BD	0.88	0.92	1

Macro Average	0.91	0.85	1.00
Weighted Average	0.93	0.76	1.00

Supplementary Figure S2. Diagnostic performance of the RF model discriminating CTRL, SZ, SA and BD. A/ Scatterplot 3D of the RF model on the test dataset for discriminate CTRL vs BD vs SA vs SZ subgroups and associated per-class performance metrics of the multiclass RF model (precision, recall and F-1 score), and their macro and weighted averages. B/ Scatterplot 3D of the RF model on the Monte Carlo simulation dataset (n = 500) for discriminate CTRL vs BD vs SA vs SZ subgroups and associated per-class performance metrics of the multiclass RF model (precision, recall and F-1 score), and their macro and weighted averages. CTRL: Control; BD: Bipolar Disorder; SA: Schizoaffective disorder; SZ: Schizophrenia.

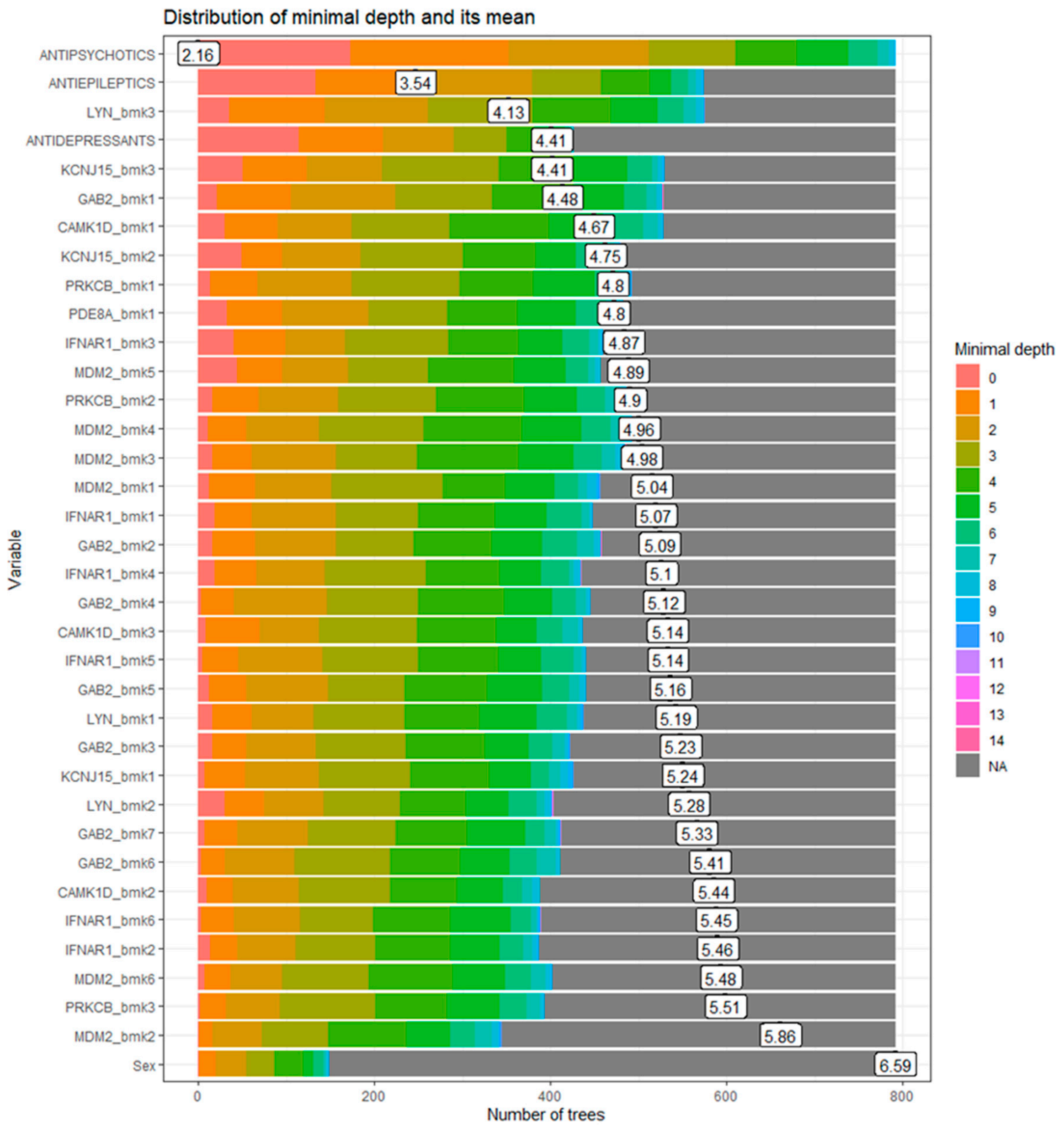

Supplementary Figure S3. Distribution of the minimum depth of variables in the RF model**.** Minimal depth value is colored according to its depth and mean value is calculated and displayed at points. The variable with the smallest mean minimum depth may be considered the most significant in the model. Antipsychotic variable emerges as the most influential variable in our model. However, RNA editing biomarkers also play a significant role, demonstrating that they are fully integrated and utilized within the model's predictive framework. Sex is the least influential variable in the RF model.

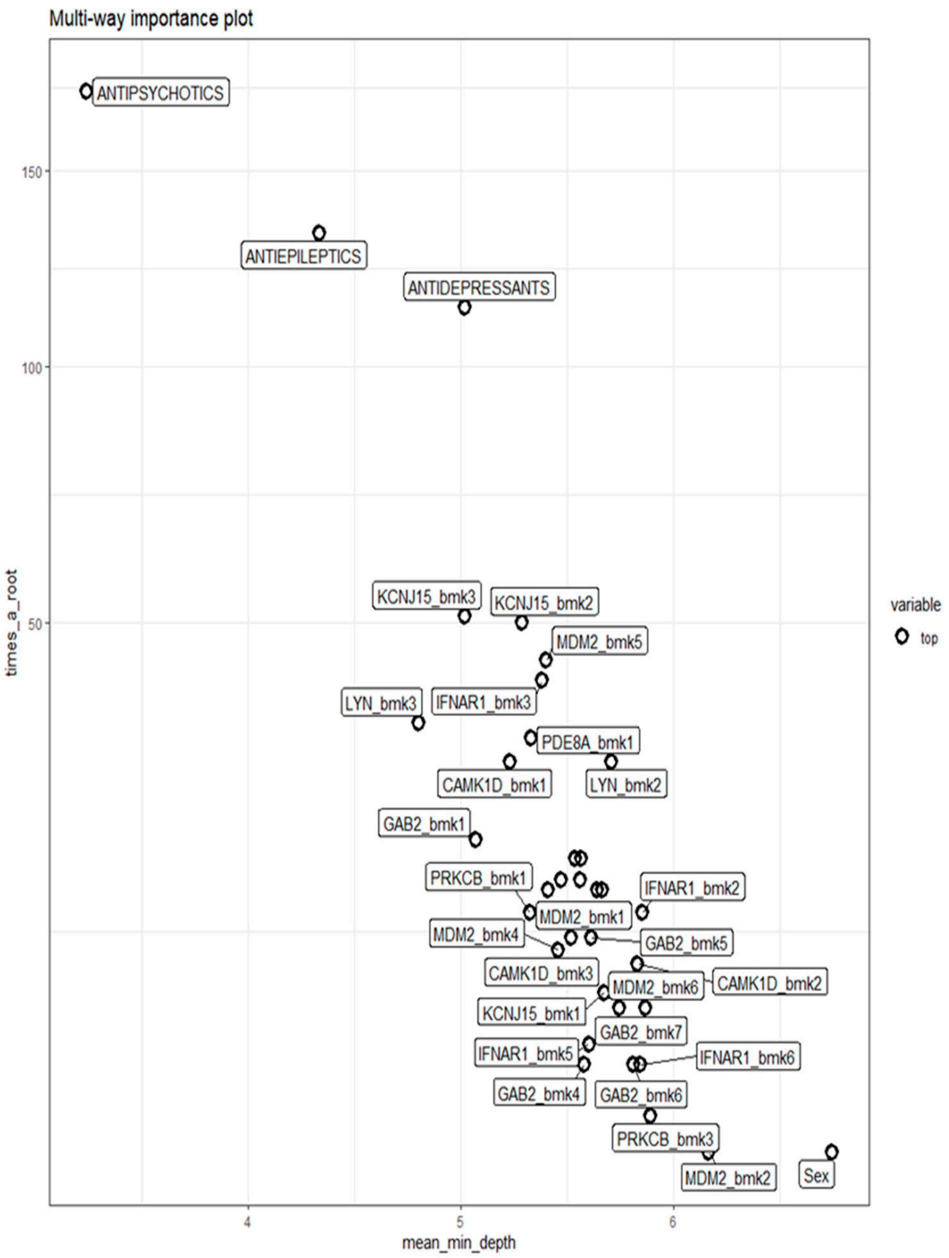

Supplementary Figure S4. Importance variable on a multi-way importance plot**.** Y-axes (times a root) show the number of trees in which the root is split for each variable, whereas the x-axes (Mean minimal depth) show the mean depth of first split for each variable. A higher frequency implies that the variable consistently provides the highest precision among the randomly selected subsets of variable used to construct each of the 1000 trees. These two metrics are visualized as a scatter plot to explore the potential interactions among variables of the RF model. Because these two measures are negatively associated, most important variables are located in the upper-left corner.

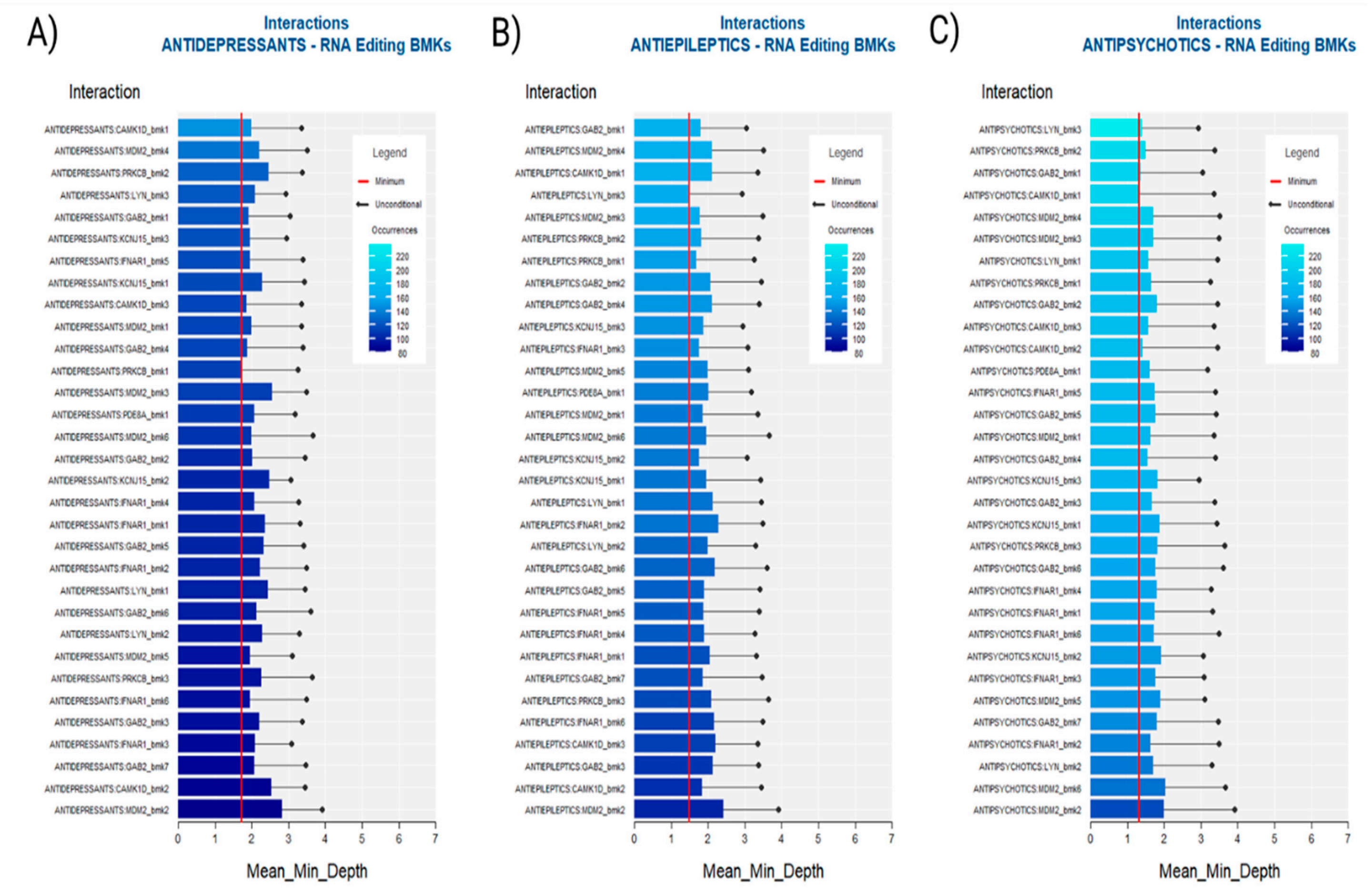

Supplementary Figure S5. Feature interaction analysis using the conditional depth. A) Mean minimal (conditional and unconditional) depth for the 32 RNA editing biomarkers interactions with antidepressants. B) Mean minimal depth for the 32 RNA editing biomarkers interactions with antiepileptics. C) Mean minimal depth for the 32 RNA editing biomarkers interactions with antipsychotics. The interactions between the 32 RNA editing variables and psychiatric treatment ATC classes have been evaluated. Specifically, it was examined how splits with RNA editing biomarkers were related to each psychiatric treatment class, such as antipsychotics, antidepressants, and antiepileptics, within the maximal subtrees. All 32 RNA editing biomarkers interact with psychiatric treatments more than 100 occur in the RF models. LYN_bmk3, CAMK1D_bmk1, GAB2_bmk1, PRKCB_bmk2 and MDM2_bmk4 are the features interacting the most with psychiatric treatments (more than 200 occur with antipsychotics).

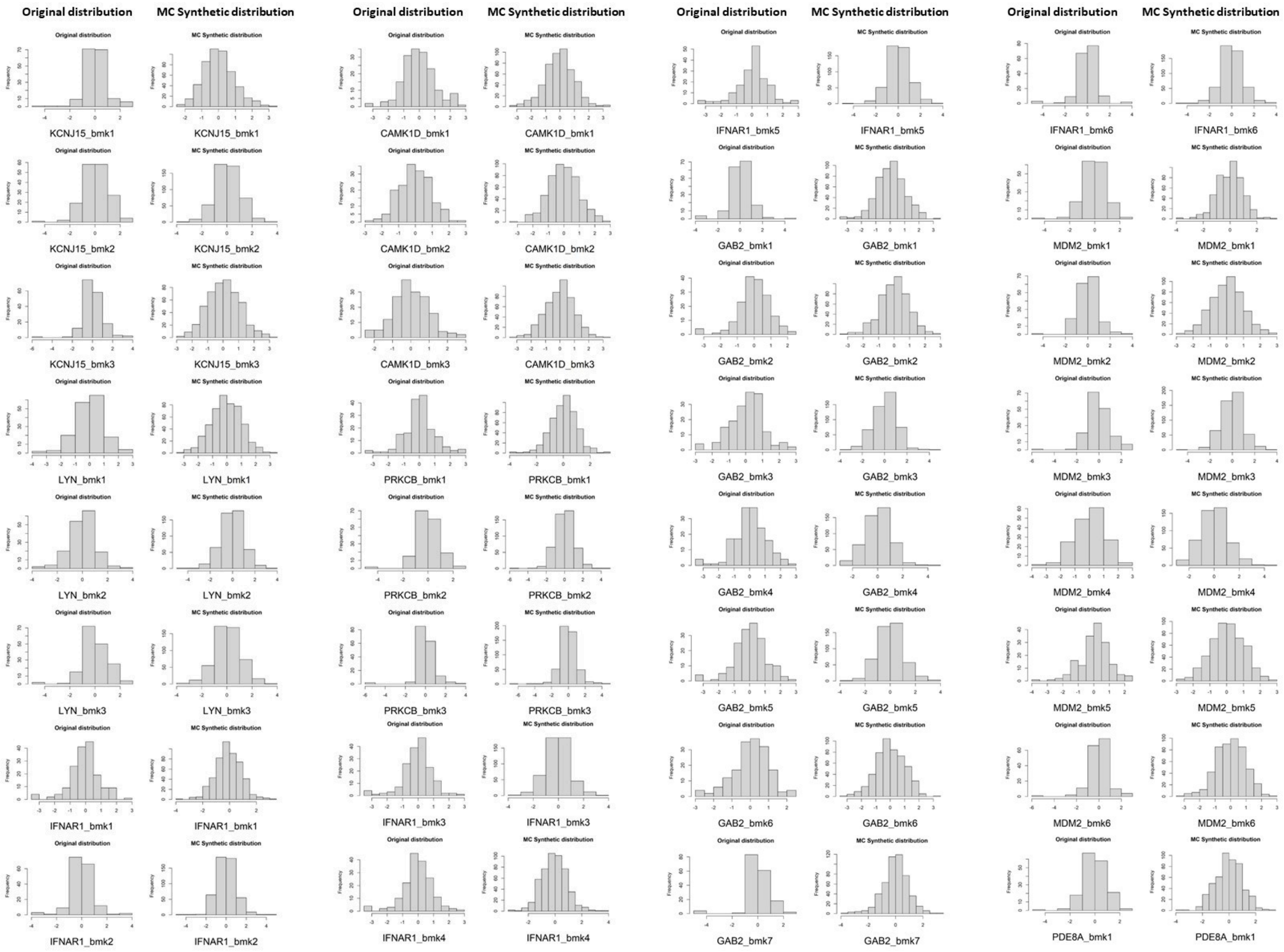

Supplementary Figure S6. Monte Carlo (MC) simulated data validation. Comparison of original data (left panels) and Monte-Carlo-simulated data distribution (right panels) across biomarkers. The Monte-Carlo (MC) data were generated through multivariate normal resampling. For each panel, Left: Distribution histogram for the normalized 32 RNA editing biomarkers values on original data; Right: Distribution histogram for the normalized 32 RNA editing biomarkers values on MC simulated data. Histograms show that the synthetic data preserve the shape, central tendency, and spread characteristics of the original distributions

References:

1. Langmead, B.; Salzberg, S.L. Fast gapped-read alignment with Bowtie 2. *Nat Methods* **2012**, *9*, 357-359, doi:10.1038/nmeth.1923.
2. Li, H.; Handsaker, B.; Wysoker, A.; Fennell, T.; Ruan, J.; Homer, N.; Marth, G.; Abecasis, G.; Durbin, R.; Genome Project Data Processing, S. The Sequence Alignment/Map format and SAMtools. *Bioinformatics* **2009**, *25*, 2078-2079, doi:10.1093/bioinformatics/btp352.
3. Gentleman, R.C.; Carey, V.J.; Bates, D.M.; Bolstad, B.; Dettling, M.; Dudoit, S.; Ellis, B.; Gautier, L.; Ge, Y.; Gentry, J.; et al. Bioconductor: open software development for computational biology and bioinformatics. *Genome Biol* **2004**, *5*, R80, doi:10.1186/gb-2004-5-10-r80.
4. Team, R.D.C. R: A language and environment for statistical computing. **2010**.
5. Salvetat, N.; Checa-Robles, F.J.; Patel, V.; Cayzac, C.; Dubuc, B.; Chimienti, F.; Abraham, J.D.; Dupre, P.; Vetter, D.; Mereuze, S.; et al. A game changer for bipolar disorder diagnosis using RNA editing-based biomarkers. *Transl Psychiatry* **2022**, *12*, 182, doi:10.1038/s41398-022-01938-6.
6. Su, J.Q.; Liu, J.S. Linear Combinations of Multiple Diagnostic Markers. *Journal of the American Statistical Association* **1993**, *88*, 1350-1355.
7. Box, G.E.P.; Cox, D.R. An Analysis of Transformations. *Journal of the Royal Statistical Society: Series B (Methodological)* **1964**, *26*, 211-243, doi:https://doi.org/10.1111/j.2517-6161.1964.tb00553.x.
8. Benjamini, Y.; Hochberg, Y. Controlling the False Discovery Rate: A Practical and Powerful Approach to Multiple Testing. *Journal of the Royal Statistical Society: Series B (Methodological)* **1995**, *57*, 289-300, doi:https://doi.org/10.1111/j.2517-6161.1995.tb02031.x.
9. Kramar, A.; Faraggi, D.; Fortune, A.; Reiser, B. mROC: a computer program for combining tumour markers in predicting disease states. *Comput Methods Programs Biomed* **2001**, *66*, 199-207, doi:10.1016/s0169-2607(00)00129-2.
10. Breiman, L. Random Forests. *Machine Learning* **2001**, *45*, 5-32, doi:10.1023/A:1010933404324.
11. Kuhn, M. Building Predictive Models in R Using the caret Package. *Journal of Statistical Software* **2008**, *28*, 1 - 26, doi:10.18637/jss.v028.i05.
12. Paluszynska A, Biecek P, Jiang Y., 2022. randomForestExplainer: explaining and visualizing Random Forests in terms of variable importance. R package version 0.10.1. https://CRAN.R-project.org/package=randomForestExplainer.
13. McCracken, D.D. The Monte Carlo Method. *Scientific American* **1955**, *192*, 90-96, doi:10.1038/scientificamerican0555-90.
14. Venables WN, Ripley BD (2002). Modern Applied Statistics with S, Fourth edition. Springer, New York. ISBN 0-387-95457-0, https://www.stats.ox.ac.uk/pub/MASS4/.
